# Highly Sensitive and Full Range Detectable Humidity Sensor using PEDOT:PSS, Methyl Red and Graphene Oxide Materials

**DOI:** 10.1038/s41598-019-51712-w

**Published:** 2019-10-23

**Authors:** Gul Hassan, Memoon Sajid, Changhwan Choi

**Affiliations:** 1 Division of Materials Science and Engineering, Hanyang University, Seoul, 04763 Korea; 2 Department of Electrical Engineering, Ghulam Ishaq Khan Institute of Engineering Sciences and Technology, Khyber Pakhtunkhwa, 23460 Pakistan

**Keywords:** Electronic properties and materials, Sensors and biosensors

## Abstract

Single transducer with humidity sensing materials has limitations in both range and sensitivity, which cannot be used to detect the full range of humidity with consistent sensitivity. To enlarge range and improve sensitivity in the all range relative humidity (RH), we propose a highly sensitive and full range detectable humidity sensor based on multiple inter-digital transducer (IDT) electrodes connected in series with poly(3,4-ethylenedioxythiophene) doped poly (styrene sulfonate) anions (PEDOT: PSS), C_15_H_15_N_3_O_2_ (Methyl Red), and graphene oxide (GO) thin films as the active sensing materials. The humidity sensor with single active material has a limit in the detecting ranges, where the GO, PEDOT: PSS, and Methyl Red materials have sensing responses of 0 to 78% RH, 30 to 75% RH, and 25 to 100% RH, respectively. However, a humidity sensor using combined three active materials can respond to much wider range of RH with high sensitivity, where the IDTs and the active regions were prepared using ink-jet printing and spin coating, respectively. This proposed sensor can detect a full range of 0% RH to 100% RH. The response and recovery times are 1 sec and 3.5 sec, respectively. Our single sensing device using multiple IDTs connected different active materials in series can overcome the limitations of single transducer based sensor for the high performance sensor applications.

## Introduction

Relative humidity (RH) sensing has garnered great attention due to versatile aspects of monitoring and controlling humidity for medicines, other sensitive products like food, human health, industry, and even in space environments^1–10^. Humidity sensors are designed to detect the amount water content in the surrounding environment by monitoring change in various parameters such as resistance, capacitance, refractive index, inductance, piezoelectric response and so on^11,12^. In particular, resistive and capacitive sensors have received high interest because of their low cost, facile fabrication, environment-friendly nature, and seamless integration with electronic circuits enabling a direct electrical readout^13^. Research on humidity sensors focuses on improving response and recovery times, sensitivity, and detection range. To achieve these target parameters for certain customized applications, different approaches have been proposed by designing different sensor structure^14,15^, changing active materials^16,17^ and adopting different fabrication technologies^3^.

Different kinds of materials have been studied for humidity sensing using ceramics^18^, polymers^19^, organic and inorganic semiconductors^20^, composites^21,22^ and oxides^23^. Although some humidity sensors were previously demonstrated with some advantages such as low cost, easiness to use, and low operational temperature with long lifetime, low sensitivity and limited detection range are still main concerns. To overcome these challenges, researchers are in the development using different materials and fabrication technologies^3^, but still further investigations are needed. Particularly, implementation of alternative materials can be a great momentum to improve sensitivity and detection range^24,25^. However, research is still lack to achieve the cost effective and full range detectable humidity sensor.

**Figure 1:**
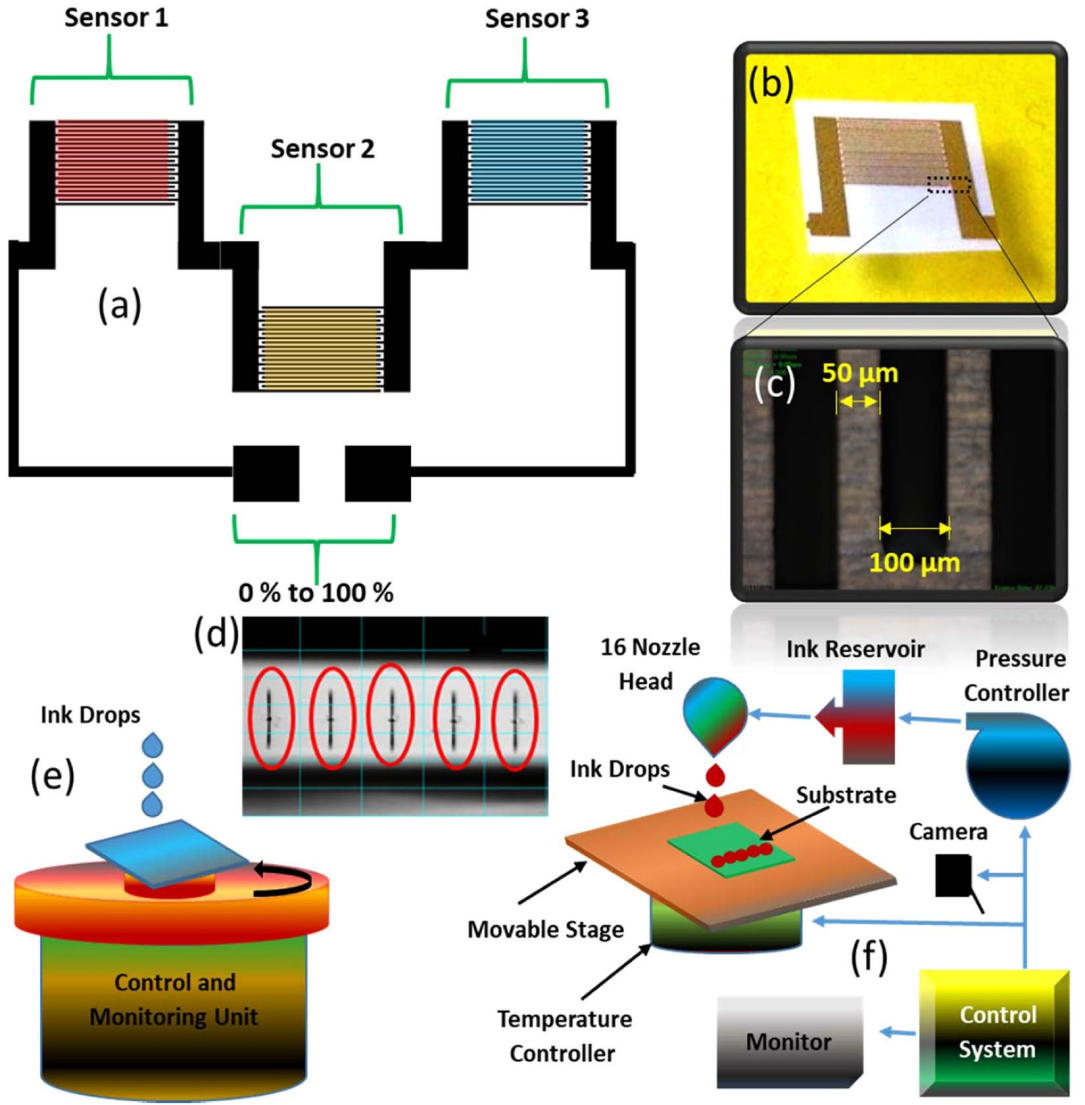
(**a**) Schematic sensor image, (**b**) single fabricated inter-digital electrode, (**c**) optical image of the inter-digital electrode fingers, (**d**) stable wave shape of the ink-jet printer DMP-3000 print head, (**e**) schematic cartoon of spin coating for the active materials, and **(f**) schematic image of the ink-jet printer DMP-3000.

In this work, we demonstrated a highly sensitive and full range detectable humidity sensor based on multiple inter-digital transducer (IDT) electrodes connected in series using graphene oxide (GO), poly(3,4-ethylenedioxythiophene) doped with poly (styrene sulfonate) anions (PEDOT: PSS), and C_15_H_15_N_3_O_2_ (methyl red) as the active sensing materials. Sensing ranges significantly depend on active materials. Sensor using GO thin film showed linear sensing response towards low humidity levels from 0% RH to 75% RH. For PEDOT: PSS-based sensor, it linearly responded to mid-range humidity levels from 25% RH to 75% RH. Sensor with methyl red-based active region showed linear sensing response towards higher humidity levels from 25% RH to 100% RH. To maximize sensing range further, these three active regions were connected in series to fabricate a single humidity sensing device enabling full range detection of relative humidity from 0% RH to 100% RH with high sensitivity. The three IDTs were fabricated by ink-jet printing as shown in Fig. 1 and the active regions were deposited via spin coating. The response and recovery times were also excellent with values of 1 sec and 3.5 sec, respectively.

## Materials and Methods

### Inter-digital transducer (IDT) electrodes

Inter-digital typed electrodes were used as the transducing elements in the proposed humidity sensing device. IDT are expected to provide higher sensitivity by efficiently detecting any change in active film characteristics on all electrode fingers. It is similar to a combination of two electrode chemristors connected in parallel with an output equivalent to their net effect. The finger width and spacing of each IDT were 100 µm and 50 µm, respectively, as shown in Fig. 1c. The spacing between the fingers was optimized to attain higher sensitivity^26^. The schematic of electrodes was firstly designed as shown in Fig. 1a and was loaded into DMP-3000 and the silver ink was loaded into the cartridge having 16 nozzles with 1 pico-liter drop size. The IDTs were fabricated through commercially available Dimatix material inkjet printer (DMP-3000). The DOD printer ejects droplets by applying voltage, which changes the volume of the channel to eject the ink from the nozzles. For the uniform and proper fabrication, the print head wave shape of DMP-3000 printer should be well-controlled and stable, which was set at 27 V as shown in Fig. 1d. The drop size was uniform, well-shaped, and very stable. The IDTs fabricated on the flexible polyethylene terephthalate (PET) substrate are shown in Fig. 1b. The thickness of the deposited silver electrode was 500 nm. The printed electrodes were sintered at 120 °C for 90 minutes^27^. Figure 1e schematically depicts the spin coating technique for active material deposition while the DMP-3000 material printer for IDTs fabrication is shown in the Fig. 1f.

### Sensing layer materials and fabrication

The active or sensing layer is the most important part of the humidity sensor. The detection of humidity or water vapors in the surrounding environment using the sensing layer can be achieved by monitoring change in resistance or capacitance. All active materials aforementioned exhibit resistance changes in response to variation in the amount of surrounding RH. The GO dispersion in H_2_O with 2 mg/ml, PEDOT: PSS in paste form, methyl red in powder form, and dimethylformamide (DMF) solvent were purchased from Sigma Aldrich, Korea. The 10 weight percent (wt %) methyl red was dissolved in DMF using bath sonication for 2 hours at 30 °C (at lower temperature, less than 15 °C) and it became as gel^26^. PEDOT: PSS paste was diluted by 10 wt% in de-ionized water, followed by stirring for 1 hour at room temperature. GO solution was used without further processing. Then, these materials were independently deposited over different sets of silver IDTs using spin coating to complete multiple active regions. The PET substrate was treated with UV-ozone plasma for 5 minutes just before deposition of each active layer thin film. UV surface treatment removes impurities and improves the hydrophilicity of the surface, leading to better adhesion of the active layer material to the surface. The treatment also results in production of hydroxyl ions on the surface that helps the materials to temporarily bond to the surface and allow homogenous thin film fabrication. All films were spin-coated at 2200 rpm and were cured for 2 hours at different temperatures for the proper sintering such as 120 °C for graphene oxide, 100 °C for PEDOT: PSS, and 130 °C for methyl red.

### Characterization

**Figure 2:**
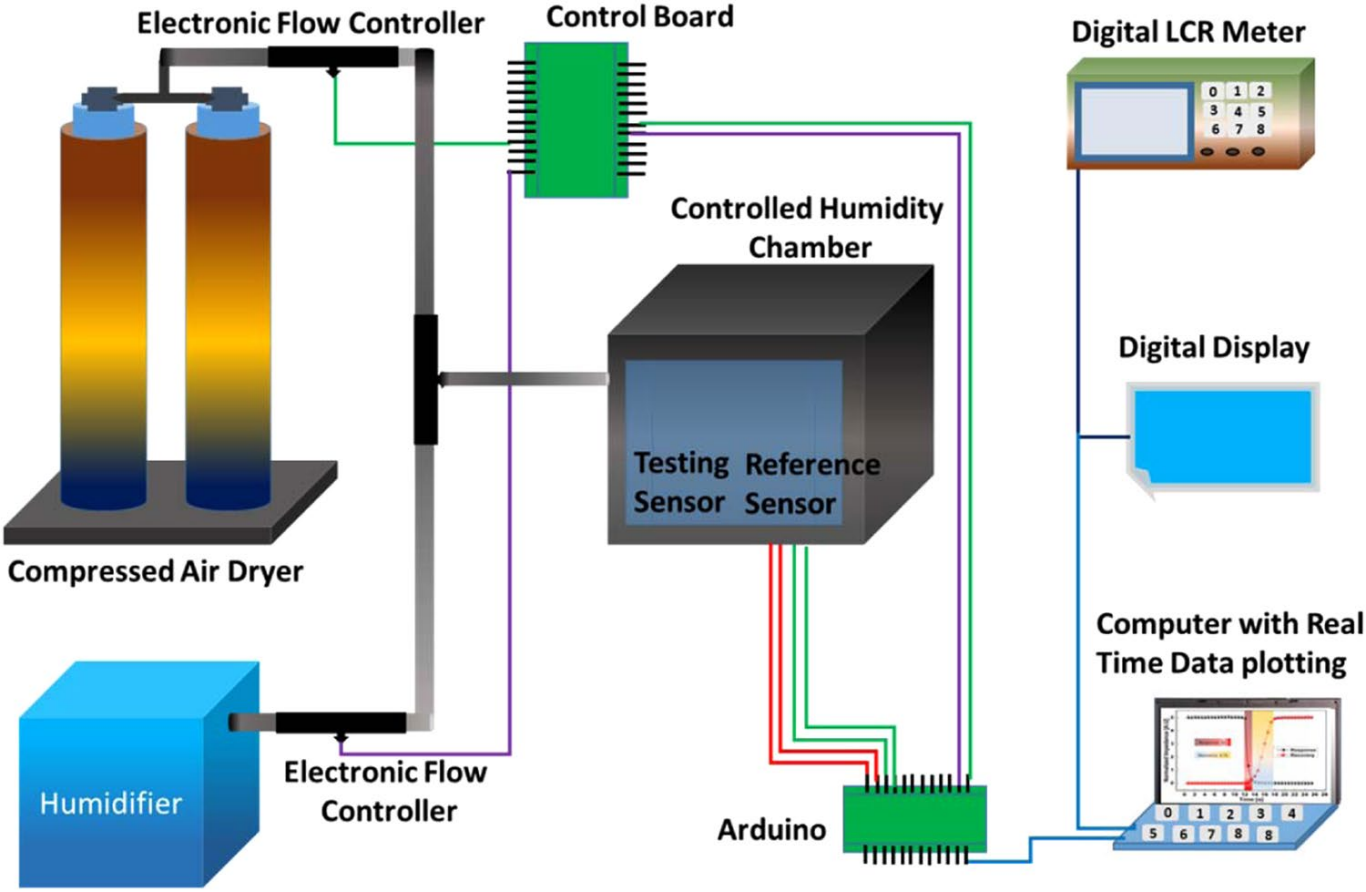
Schematic set-up of characterizing response against relative humidity change in our sensors.

The surface morphology of the sensing layers was analyzed using FE-SEM (JEOL JSM-7600F). NV-2000 (Universal) non-contact surface profiler was employed in phase shifting interferometry (PSI) mode to investigate the proper fabrication of IDTs. The humidity sensing characterization setup is presented as shown in Fig. 2.

RH was controlled inside the enclosed environmental chamber where the reference sensor (HTU-21D) and the sensor being evaluated were placed side by side for data recording. EDC-1630 digital LCR meter was used to record the impedance and resistance data of the fabricated sensors. The real time data curves were also plotted on an oscilloscope through a DAQ circuit. The airtight humidity chamber can cover full range of relative humidity from 0% to 100% RH. The RH inside the chamber was maintained using automatic feedback controller. Compressed air dryer along with dry nitrogen were used to decrease the level of RH while a desktop humidifier was used to increase the humidity. The flow rate was controlled through electronic valves and the real time data was displayed on an LCD along with data logging and plotting on a computer. The humidity level was changed by ~5 ± 1% RH per step and was maintained until the real time readings of the device under test (DUT) were stable. The humidity responses of the proposed devices using 4 different active layers PEDOT: PSS, methyl red, GO thin film, and the series combination of all three materials were taken for the range of 0% RH to 100% RH and the temperature was maintained at ~21 °C for the whole experiment. The impedance response of each sensor was measured for a full humidity range from 0% RH to 100% RH and the test frequency was kept constant at 1 kHz. The reading was allowed to become stable before incrementing to the next step and an average of 20 points was taken for every specific value at 5% RH interval.

## Results and Discussion

**Figure 3:**
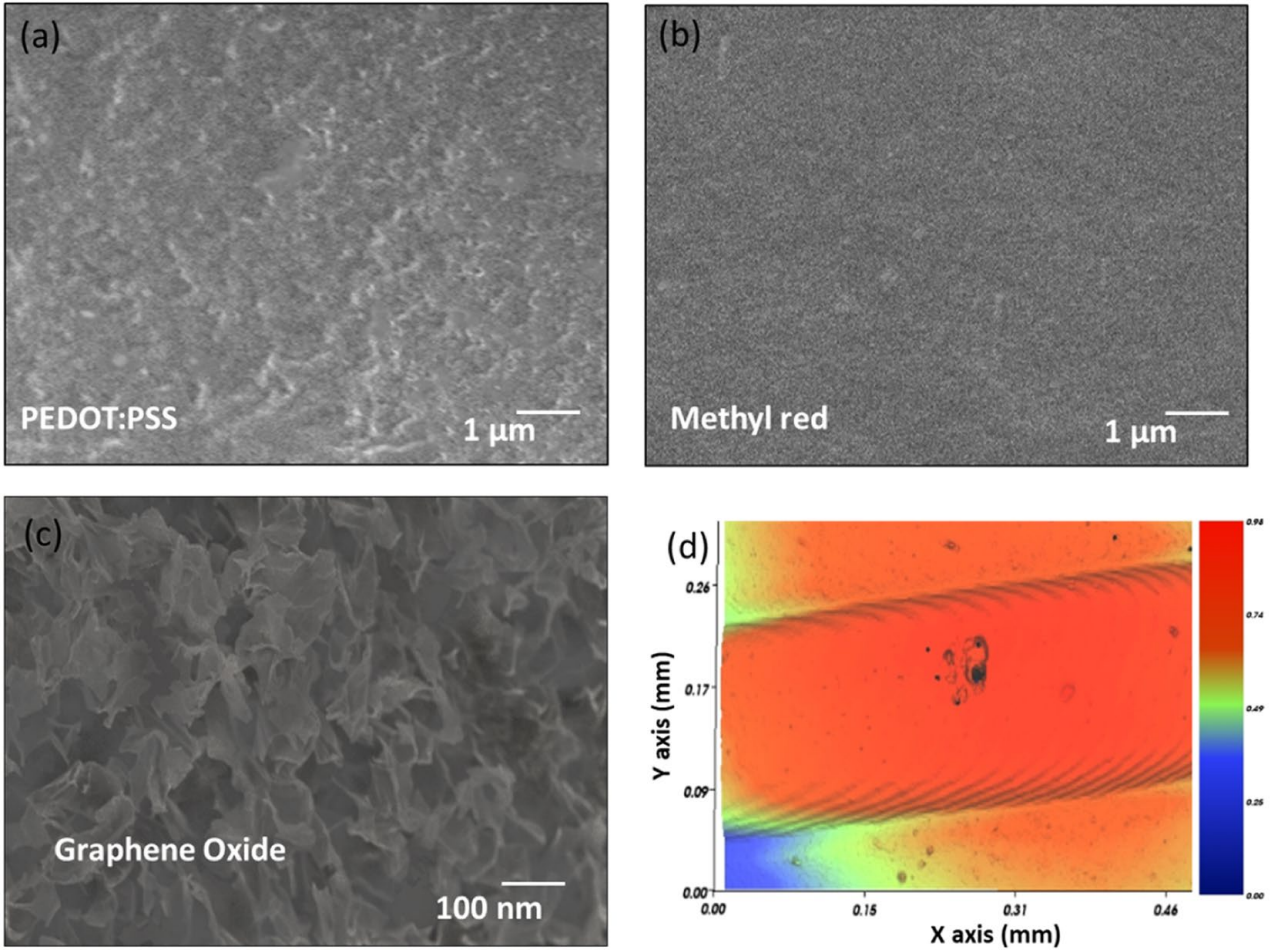
Surface morphology characterizations showing SEM images of (**a**) PEDOT: PSS thin film, (**b**) methyl red thin film, (**c**) GO thin film, and (**d**) 3D morphology image of an IDT electrode finger.

Surface morphologies of the active regions consisting of PEDOT: PSS, methyl red and GO thin film analyzed using FE-SEM are presented in Fig. 3, indicating that the sensing films are uniformly deposited. Uniform PEDOT: PSS and crack-free methyl red without any visible defects are shown in Fig. 3a,b, respectively. GO thin film turns out to be highly porous pointing towards a high surface area to volume ratio for maximum adsorption of the water molecules as shown in Fig. 3c. All the sensing films were uniformly deposited and well-cured without any visible irregularities. In addition, 3D profile of an IDT electrode finger shows a clear edge and uniform height profile as shown in Fig. 3d.

**Figure 4:**
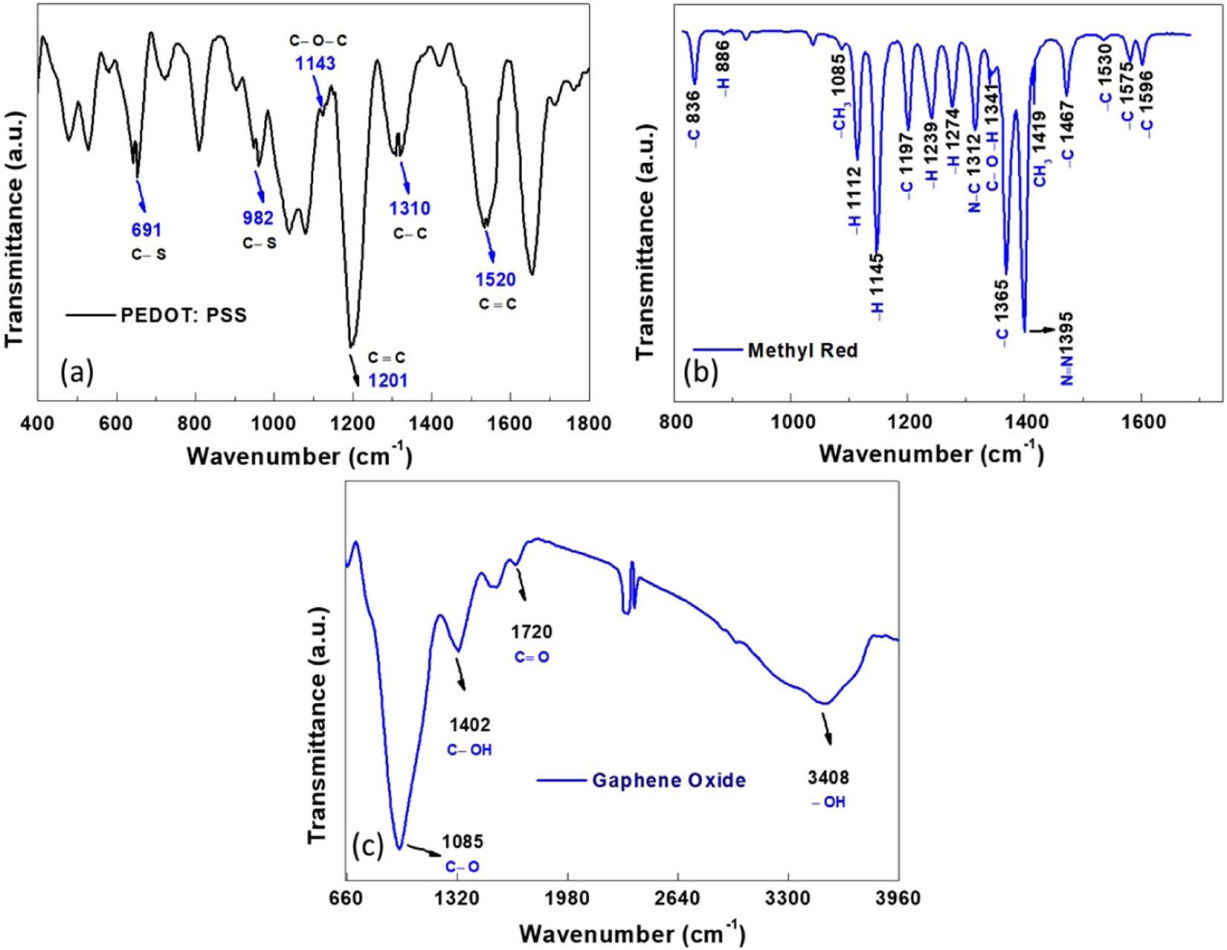
FTIR characterizations (**a**) PEDOT: PSS, (**b**) methyl red and (**c**) Graphene Oxide.

The chemical composition of PEDOT: PSS, methyl red and GO thin films were investigated by using FTIR spectroscopy as shown in Fig. 4. The Fig. 4a shows the FTIR spectra of PEDOT: PSS in the range of 400 cm^−1^ to 1800 cm^−1^. The IR bands of 1520 cm^−1^, 1310 cm^−1^ and 1201 cm^−1^ are mainly due to C = C and C – C stretching of quiniod structure of the thiophene rings and sulfonic acid group of PSS. The band at 1143 cm^−1^ is due to C – O – C bond stretching in the ethylene dioxide units while the bands at 982 cm^−1^ and 691 cm^−1^ are attributed to C – S stretching mode^28^. These bands indicate the pure nature of the PEDOT: PSS without any contamination even after deposition.

The FTIR spectrum of methyl red is shown in Fig. 4b, here bands at 886 cm^−1^, 1112 cm^−1^, 1145 cm^−1^, 1239 cm^−1^ and 1274 cm^−1^ are the Ring−H stretching modes while the seven bands at 836 cm^−1^, 1197 cm^−1^, 1365 cm^−1^, 1467 cm^−1^, 1530 cm^−1^, 1575 cm^−1^ and 1596 cm^−1^ are the Ring−C stretching modes attributed to benzene rings in methyl red. Band at 1395 cm^−1^ shows a strong characteristic N = N stretching and a weak band at 1419 cm^−1^ is assigned to the CH_3_ symmetric deformation. The band at 1085 cm^−1^ is CH_3_ rocking and band at 1341 cm^−1^ is attributed to C − O – H while 1312 cm^−1^ band is amine N − C^29^. These bands suggest that methyl red thin film has no impurities and contamination. Figure 4c shows the FTIR spectra of GO thin film in the range of 660 cm^−1^ to 3960 cm^−1^and the spectrum of GO has a well-built and broad − OH stretching vibration band at 3408 cm^−1^, carboxyl C = O stretching band at 1720 cm^−1^, C − OH deformation vibration band at 1402 cm^−1^ and C − O stretching vibration at 1085 cm^−1^ because of extensive oxidation^30^. These bands also conform the pure nature and composition of GO without impurities.

**Figure 5:**
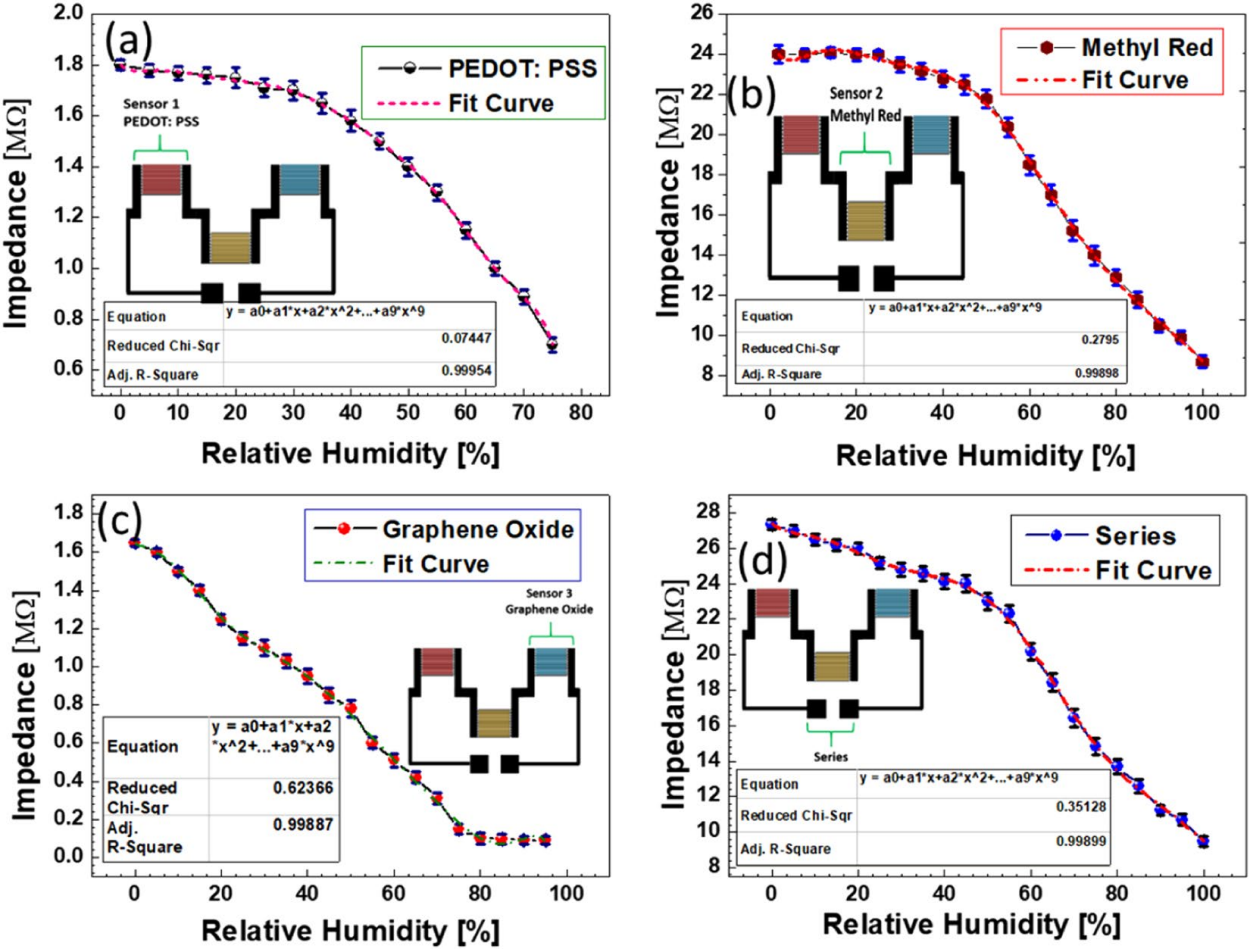
Impedance response of the sensors with (**a**) PEDOT: PSS, (**b**) methyl red, (**c**) GO thin film, and (**d**) combination of the three active sensing layers in series.

Figure 5 represents the impedance responses of each individual sensing device as well as device with all three materials combined in series. The resultant impedance change corresponds to the change in RH, where the impedance of all four sensors decreases with increasing humidity levels. For the sensor with PEDOT: PSS active area, there is small change in impedance (1.8 MΩ to 1.7 MΩ) at the low range levels from 0% to 25% RH. However, further increasing the RH, there is a quick drop in impedance as shown in Fig. 5a. These results suggest that the PEDOT: PSS based active region has low sensitivity towards low humidity levels and higher sensitivity towards high level of surrounding environment humidity. PEDOT: PSS is a conductive polymer in nature^31^ and its resistance drops in response to increase in humidity but this drop is very small at lower RH as compared to higher RH levels. This result can be attributed that the water molecules are not directly participating in the generation of ionic current, but the facilitation of electronic current flow becomes strong by providing wider, shorter, and more connected paths to current^13^. Adsorption of water molecules in PEDOT: PSS causes swelling and dissociation of PEDOT and PSS bonds leading to increased bond energy between PEDOT chains. Since the amount of physically- adsorbed water molecules is very low at lower humidity levels, there is almost no swelling or dissociation and the change in impedance is very low. In this case, the change in capacitance as result of the physisorbed water molecules dominates the resultant change in impedance of the sensor. At higher humidity levels, hydroxyl groups in water molecules can form a coulombic bond with PEDOT increasing its positive charge density. This can cause PEDOT: PSS to change from a benzoid structure to a quiniod structure that has straighter chains and facilitates current flow resulting in the impedance to drop. Finally, after 80% RH, there is no further visible change in impedance inducing the saturation point, which is defined as a maximum detection limit of the PEDOT: PSS based active layer. The impedance of every individual sensor element can be expressed by the following Eq. (1)^21^

1 $$Z=\frac{1}{2\pi fC}+{R}_{s}$$

where R_s_ is the sample resistance, *f* represents the test frequency (1 KHz), and *C* is the capacitive factor of the equivalent circuit of the sensor. From Eq. (1), it is apparent that decrease in resistance or increase in capacitance leads to the decrease of overall impedance of the sensor.

For the sensor with methyl red-based active layer, it has a low sensitivity towards lower levels of RH considering no significant change in impedance till 25% RH as shown in Fig. 5b. Methyl red is a non-conductive material resulting in no flow of electronic current under normal condition. This indicates that low levels of RH cannot considerably affect the overall impedance of the sensor. However, at higher levels of RH, change in capacitance due to the physisorbed water molecules affects impedance change similar to PEDOT: PSS case. This capacitance changes in case of methyl red mainly depends upon the polarization^32^, dielectric permittivity of the sensing layer material, the electrode geometry^33^ and gap between the electrodes^26^. The polarization is mainly due to electronic and ionic polarizability, and dipoles created in the active region upon adsorption of hydroxyl ions and water molecules. The ions and dipoles are formed due to dissociation of molecules and charge transfer complexes whereas the electronic polarization occurs due to relative dislocation or displacement of electrons in methyl red. The relationship between change in capacitance and dielectric permittivity is shown in the following Eq. (2)^4^

2 $$\frac{{C}_{s}}{{C}_{g}}=(\frac{{\varepsilon }_{w}}{{\varepsilon }_{d}})n$$

where *ε*_*w*_ and *ε*_*d*_ are the permittivity values of dielectrics at wet and dry states, respectively. As we discussed above, at higher levels of humidity the capacitance change dominates the impedance drop of sensor. This change in impedance is due to the high concentration of dipoles, ions, holes, and electrons created at high humidity levels. In addition, the increase in overall polarization contributes to this change in methyl red. The high concentration of water molecules adsorbed in the thin film induces the increase in capacitance as the equivalent resulting dielectric permittivity of methyl red increases by the absorption of water molecules. The impedance near- linearly drops form 24 MΩ to 8.7 MΩ for increasing humidity levels from 25% RH to 100% RH for methyl red-based sensor. Unlike two cases aforementioned, for sensor with GO thin film-based active layer, there is a visible change in impedance at lower humidity levels as shown in Fig. 5c. At low % RH levels water molecules are chemisorbed and physisorbed into the active sites of GO surface (vacancies or hydrophilic groups) through hydrogen bonding. The hooping transfer of carriers between the adjacent hydroxyl groups due to water molecules leads to the decrease of resistance and therefore the overall sensor impedance decreases. Another factor resulting in higher sensitivity of GO thin film-based sensor towards lower humidity levels is the physical morphology of the thin film with a highly rough surface, which allows the water molecules to readily get trapped inside. For the sensor with multi-elements combined in series, the impedance result shows the capability to detect humidity for a full detection range from 0% RH to 100% RH as shown in the Fig. 5d. The effective impedance of the sensor is the sum of the impedance of all three individual sensors. The resulting impedance curve apparently shows slightly different response behaviors with different slopes. This enables for the suitable implementation in various applications and facile integration with read-out circuits. The overall impedance changes from 27.5 MΩ to 9.48 MΩ in response to changing humidity levels from 0% RH to 100% RH. This gives us a sensitivity of around 180 kΩ/% RH.

**Figure 6:**
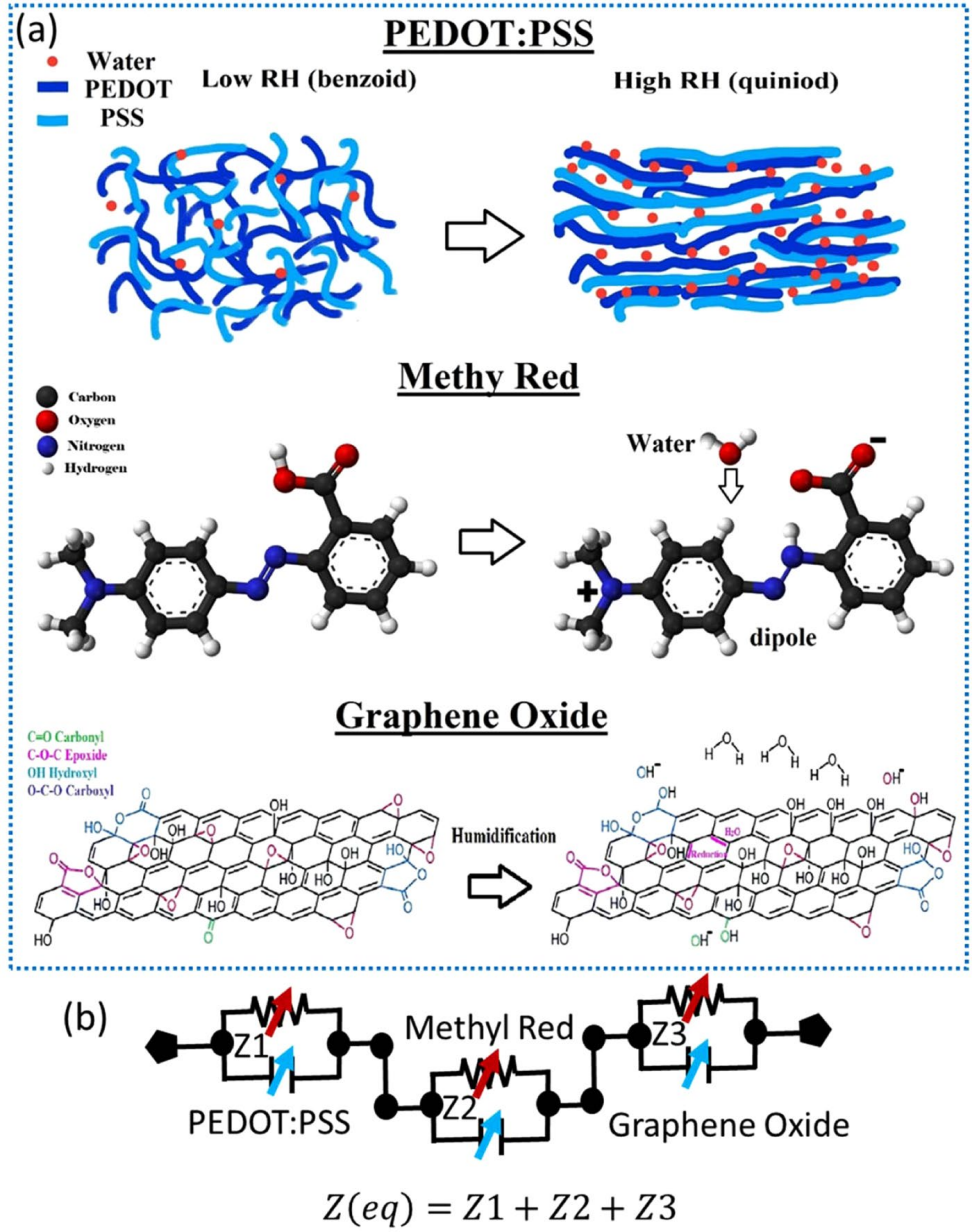
(**a**) Schematics of the sensing mechanism of PEDOT: PSS, methyl red and graphene oxide (**b**) simplified equivalent circuit of the series combination of three sensors.

The schematic illustration of the sensing mechanisms is presented in the Fig. 6a. By further increasing the humidity level, the physisorbed water molecules get ionized under the applied electrostatic field to produce a large number of hydronium ions (H_3_O^+^) which are charge carriers and leads to decrease of the impedance further. Another factor resulting in higher sensitivity of GO thin film-based sensor towards lower humidity levels is the physical morphology of the thin film that has a highly rough surface allowing the water molecules to readily get trapped inside. The sensor with GO thin film-based active layer exhibits linear decrease in impedance from 1.65 MΩ to 0.15 MΩ as increasing the humidity levels from 0% RH to 75% RH. Upon further increase in the RH, the physisorbed water acts as layer like liquid and thus no further visible change occurs in impedance of the sensor producing the saturation point. As it can be observed, the responses of individual sensor aforementioned are different in terms of detection range and sensitivity towards different humidity levels. GO thin film-based sensor has high sensitivity in range from 0% RH to 75% RH. PEDOT: PSS active layer-based sensor detection range is from 25% RH to almost 80% RH. The sensor with active region based on methyl red thin film detects humidity with high sensitivity from 25% RH to 100% RH. To detect the full range of relative humidity from 0% RH to 100% RH, the response of the above three sensors was to be ideally merged. Therefore, we connected the three sensors in a series combination producing a single sensor with multiple sensing elements having different active sensing layers and different detection ranges. The simplified equivalent circuit of the resulting sensor is presented in Fig. 6b.

**Figure 7:**
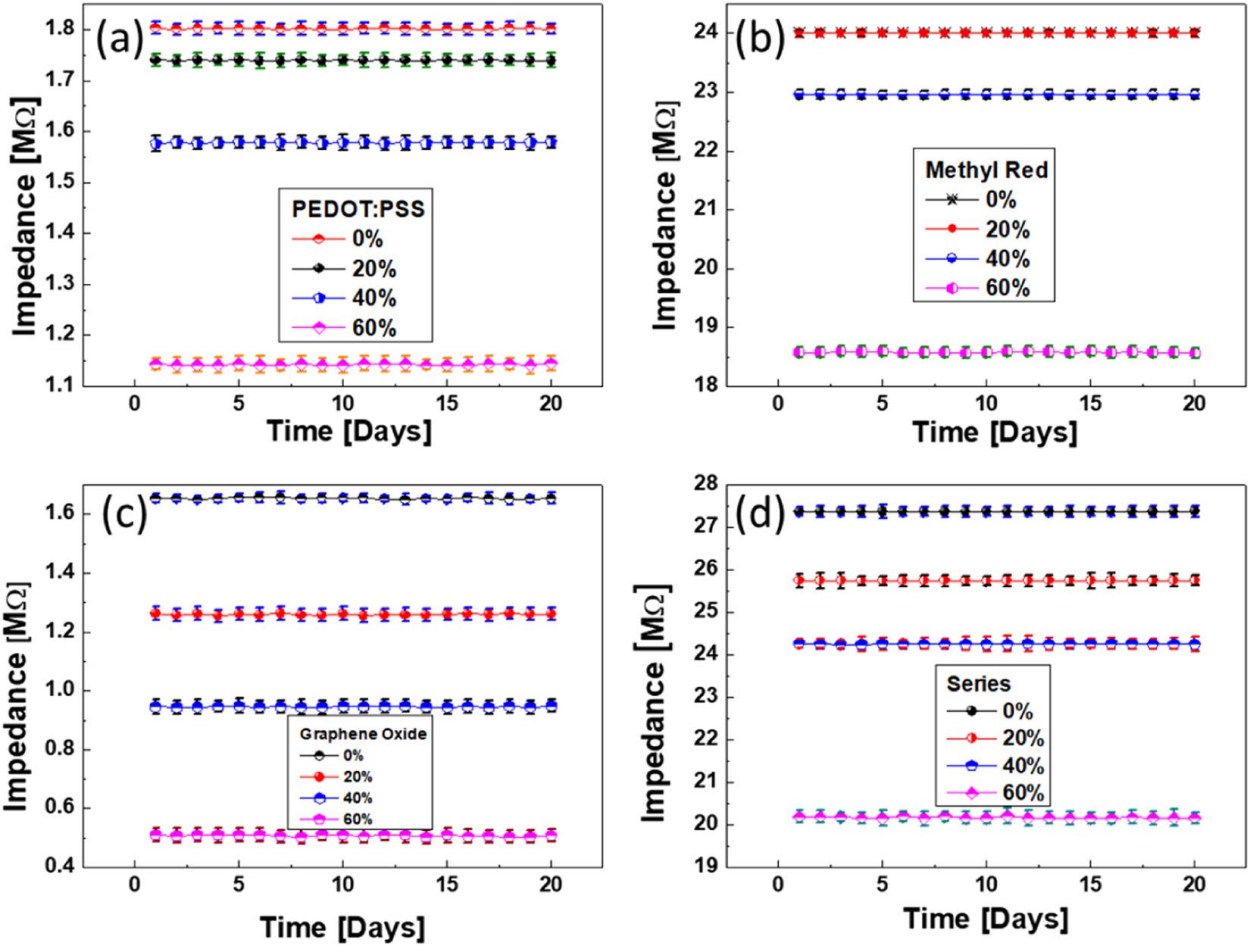
Stability responses at different humidity levels of (**a**) PEDOT: PSS, (**b**) methyl red, (**c**) graphene oxide, and (**d**) whole device (series combination of PEDOT: PSS, methyl red and graphene oxide), respectively.

Furthermore, each sensor and the whole device were tested for their stability and robustness by recording the impedance at four different humidity levels during consecutive 20 days, where the devices were stored under ambient conditions to prevent from harsh external environment. Figure 7a shows the stability of PEDOT: PSS sensor at 0%, 20%, 40% and 60% RH with an average error < 2.1%. Similarly, Fig. 7b shows the stability of methyl red at same aforementioned humidity levels with average error < 1.8%. Figure 7c shows the stability of graphene oxide sensor with average error < 2%. Finally, the whole device (series combination of PEDOT: PSS, Methyl Red and Graphene Oxide sensors) was tested at different humidity levels (0%, 20%, 40% and 60% RH) and found that the device shows stability with average error of 2.2%, which is still better than most of the high-end devices as shown in Fig. 7d.

**Figure 8:**
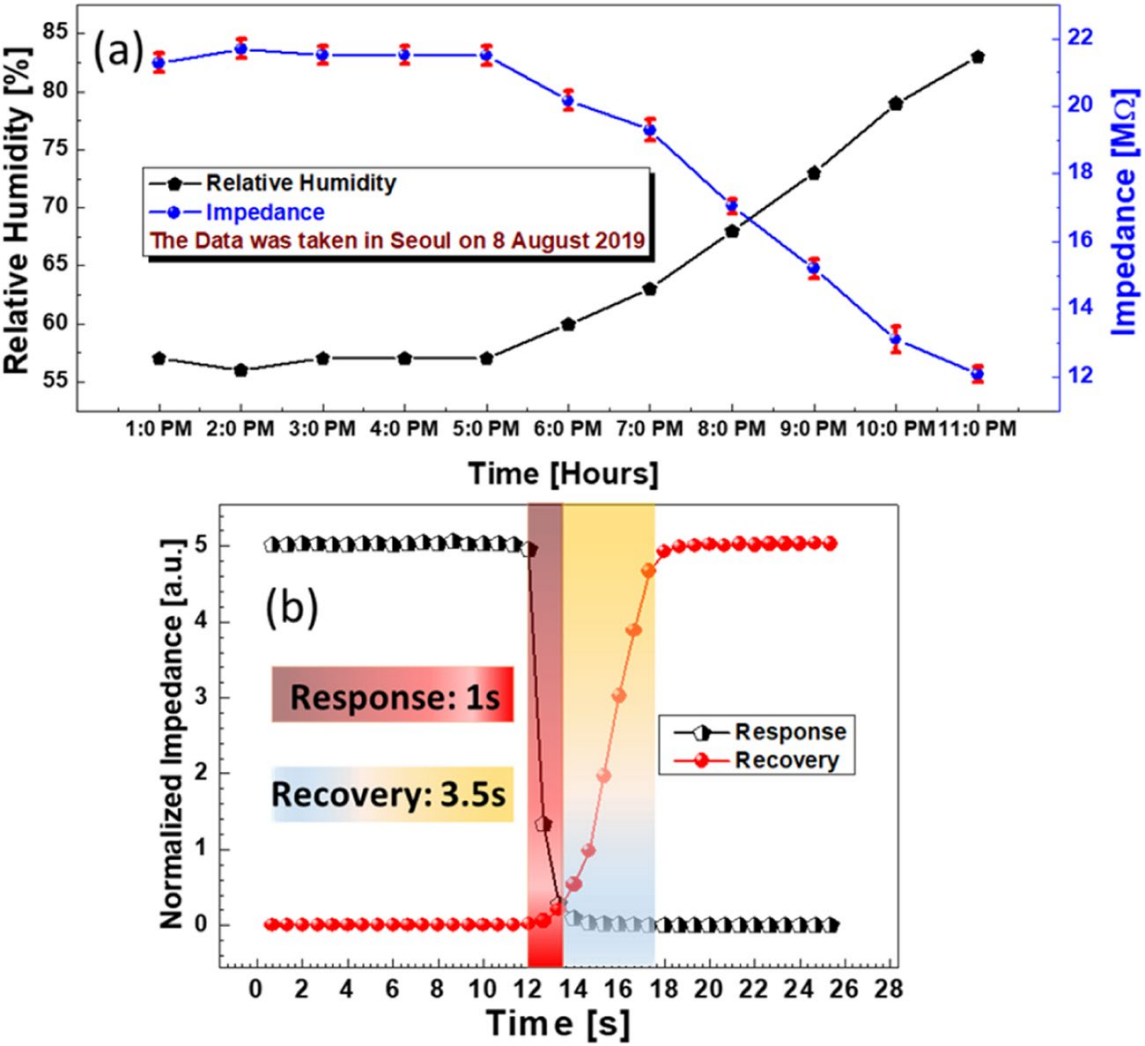
(**a**) Impedance response data of the sensor in actual environment and its stability (the data was taken in Seoul on 8 August 2019) (**b**) Transient response and recovery time curves of the proposed humidity sensor.

Afterward, the device was tested in the open natural environment to check its real time application and stability as shown in Fig. 8a. The sensor was placed along commercial sensor in open natural environment and the actual data was record after every hour from 1:00 PM to 11:00 PM in Seoul, Korea, on 8 August 2019 as shown in Fig. 8a. The device shows stable performance, indicating that it is suitable for real-time application and environment. The transient response of the resulting sensor was also evaluated using one stream of compressed dry air and a second stream of highly humid air. The electronic valves were opened and closed with pulses to expose the active sensing area directly to the incoming streams. The real time data was recorded and plotted in the computer. The response time was recorded to be ~1 second while the recovery time was around ~3.5 seconds as shown in Fig. 8b. The device is capable to respond quickly to sudden changes in humidity levels, indicating that the devices can be implemented in real life testing and applications for the quantitative humidity measurement in full range with a fast response time.

## Conclusions

Highly sensitive humidity sensor enabling to detect all range of RH level was successfully demonstrated using IDT electrodes connected in series utilizing PEDOT: PSS, methyl red and GO thin film as active layer materials. The conductive polymer PEDOT: PSS based sensor portion responds linearly to mid-range of humidity levels from 25% RH to 75% RH, which is linked to simple adsorption inducing resistance drop due to increase in electronic current. The methyl red-based active region shows linear sensing response towards higher humidity levels from 25% RH to 100% RH, which is correlated with ionic and dipole based polarization due to the physisorbed water molecules and hydroxyl ions. The GO thin film-based active region exhibits linear sensing response towards lower humidity levels from 0% RH to 75% RH and the sensing mechanism can be interpreted by chemisorption of hydroxyl ions resulting in ionic conduction. However, using the three IDTs fabricated by ink-jet printer on the flexible PET substrate as well as the active regions deposited by spin coating, sensor with combined three active sensing materials in series can achieve full sensing range of 0% RH to 100% RH. The response and recovery times were 1 second and 3.5 seconds, respectively. This approach of multiple IDTs in a single sensing device can be an alternative to single transducer-based sensing devices with limitations in nature and therefore applicable to develop for the performance sensors.
